# Functional and Survival Outcomes of Patients following the Harrington Procedure for Complex Acetabular Metastatic Lesions

**DOI:** 10.3390/curroncol29080464

**Published:** 2022-08-19

**Authors:** Andrea Plaud, Jean Gaillard, François Gouin, Aurélie Le Thuaut, Peggy Ageneau, Juliane Berchoud, Alban Fouasson-Chailloux, Vincent Crenn

**Affiliations:** 1 Clinique Chirurgicale Orthopédique et Traumatologique, Nantes Université, CHU Nantes, 1 Place Alexis Ricordeau, 44000 Nantes, France; 2 Département de Chirurgie, Centre de Lutte Contre le Cancer Léon Bérard, 69008 Lyon, France; 3 Plateforme de Méthodologie et Biostatistique, Direction de la Recherche et de l’Innovation, Nantes Université, CHU Nantes, 44000 Nantes, France; 4 Médecine Physique et Réadaptation Locomotrice, Nantes Université, CHU Nantes, 85 rue Saint Jacques, 44093 Nantes, France; 5 INSERM UMR 1229, Regenerative Medicine and Skeleton, RMeS, Nantes Université, ONIRIS, 44042 Nantes, France; 6 CRCI2NA (Centre de Recherche en Cancérologie et Immunologie Nantes-Angers), INSERM UMR 1307, CNRS UMR 6075-Team 9 CHILD (CHromatin and Transcriptional Deregulation in Pediatric Bone Sarcoma), Nantes Université, 1 rue Gaston Veil, 44035 Nantes, France

**Keywords:** acetabular lesion, bone metastasis, Harrington procedure, functional assessment

## Abstract

Background: The Harrington surgical technique makes it possible to manage complex, extensive bone lesions using pins and cement to consolidate bone for acetabular cup positioning. However, it may be associated with a high reoperation rate, and the functional results of this surgery are not precisely described in the literature. Methods: In a monocentric retrospective study including all patients operated on using the Harrington procedure associated with THA between 2005 and 2020, we aimed to assess preoperative and postoperative function, reoperation-free survival, and overall survival. Results: Functional improvement was significant for Parker scores (preoperative: 3.6 ± 2.0; 6-month follow-up: 6.6 ± 3.2; 12-month follow-up: 7.6 ± 2.1) and Musculoskeletal Tumor Society (MSTS) scores (preoperative: 31.1 ± 16.2%; 6-month follow-up: 67.7 ± 30.6%; 12-month follow-up: 82.4 ± 24.0%). Of the 21 patients included, the reoperation-free survival rate was 76.1% [CI 95%: 58.1–99.7] at six and twelve months, with the main complications being pin migration (50.0%) and infection (25%). The patient overall survival rate was 76.2% [95% CI: 59.9–96.7] at six months and 61.9% [95% CI: 59.9–96.7] at 12 months. Discussion: These results underlined significant functional improvements following a conventional Harrington procedure, with acceptable reoperation rates.

## 1. Introduction

Oncology is an exceptionally dynamic medico-surgical field in terms of research. The medical progress made in recent years has led to a marked improvement in patients’ quality of life in metastatic bone situations [1,2]. Life expectancy has also increased thanks to targeted therapies or immunotherapies. As a result, metastatic bone cancers now have a better survival prognosis [3,4,5,6], and more patients might need surgeries for these metastatic conditions [7,8]. These complex surgical procedures are decided in multidisciplinary meetings when medical treatments and radiation therapy are no longer an option.

The pelvis is the third most common site of skeletal metastases, and iliac lesions can lead to major functional deterioration, leading to complications of decubitus and a worse survival prognosis [9]. These lesions can be treated with cementoplasty [10] or total hip arthroplasty (THA) when the acetabulum is involved, but in a few cases, there is not enough bone support to position a conventional acetabular component. This situation cannot be treated with conventional techniques because of extensive bone loss, irradiated bone and soft tissues, and immunocompromised status [11]. In these complex cases, the conventional Harrington procedure using pins and cement has been described to bridge extensive iliac bone loss and to restore implant support [12,13]. Many modified Harrington procedures have been described since, using screws, various acetabular reinforcement implants, or retrograde pinning [14,15,16,17]. In every patient, the expected objective of this prosthetic procedure is the restoration of the ilio-femoral weight-bearing axis with strong anchorage of the acetabular implant, restoring mechanical support in the acetabular roof, anterior or posterior wall, even in case of pelvic discontinuity.

The final goal of the Harrington procedure is improved function and autonomy with no limitation because of pain. This procedure may also be associated with a high rate of complications in frail patients, whose prognosis for survival needs to be assessed precisely. Therefore, to refine our indications, a better understanding of both the functional impact and reoperation risk, as well as of patient survival following the Harrington procedure associated with THA, is mandatory.

We hypothesized that a Harrington procedure might improve function in operated patients. We thus aimed to compare functional scores before and after the surgery. We also aimed to assess reoperation-free survival and recorded postoperative complications. Finally, we described patient overall survival following this procedure and looked for the Katagiri prognosis score following the Harrington procedure.

## 2. Materials and Methods

### 2.1. Study Design

We carried out a retrospective, monocentric, continuous study that included all the patients treated between 2005 and 2020 in the orthopedic surgery department at Nantes University Hospital for a metastatic iliac localization using a Harrington procedure (Steinman pins and cement) associated with a THA.

#### 2.1.1. Participants 

Patients were identified within our institution patient database with a first screening using keyword requests on operating reports in the inclusion interval (263 patients) (“Harrington” or “pin” + “iliac” + “metastasis” or “pin” + “metastasis” + “acetabulum”). A secondary reading of patient records was performed on the 263 initially screened patients, leading to the inclusion of 21 cases.

#### 2.1.2. Data

Functional assessment was performed retrospectively on patient reports using Musculoskeletal Tumor Society (MSTS) scores and Parker Scores preoperatively, at six and twelve months of follow-up [18,19]. Demographic data such as age, sex, ASA (American Society of Anesthesiologists) score, anteriority, Katagiri score items (using different score versions before [20] and after 2014 [21]), and primitive cancer were recorded, as well as associated adjuvant treatments, and preoperative embolization. Surgical data were also recorded: surgery duration, bleeding, acetabular implant type, number of pins used. Patient survival, revisions and complications were recorded using the Henderson classification [22]. Retrospective radiological analysis searched for any acetabular component loosening (defined by progression of radiolucent lines on consecutive control X-rays) or pin migration (defined as a movement of ≥5 mm on consecutive control X-rays).

### 2.2. Extensive Acetabular Bone Loss Management

#### 2.2.1. Treatment Strategy

All the surgical indications were assessed using a multidisciplinary approach including an orthopedic surgeon, anesthesiologist (preoperative assessment), and oncologists for patients with an expected survival of more than six months. The patients underwent surgery due to pain, increased risk of fracture, or to re-establish the possibility of full weight-bearing. The surgery was planned with a systematic CT scan assessing bone loss and acetabular support according to the Harrington classification. The Harrington procedure (Steinman pin and cement) was associated with a THA when no other alternative implant seemed possible (such as a conventional cemented acetabular cup associated with Kerboul cross-plate or stemmed acetabular cup alone).

#### 2.2.2. Surgical Procedure

**Figure 1:**
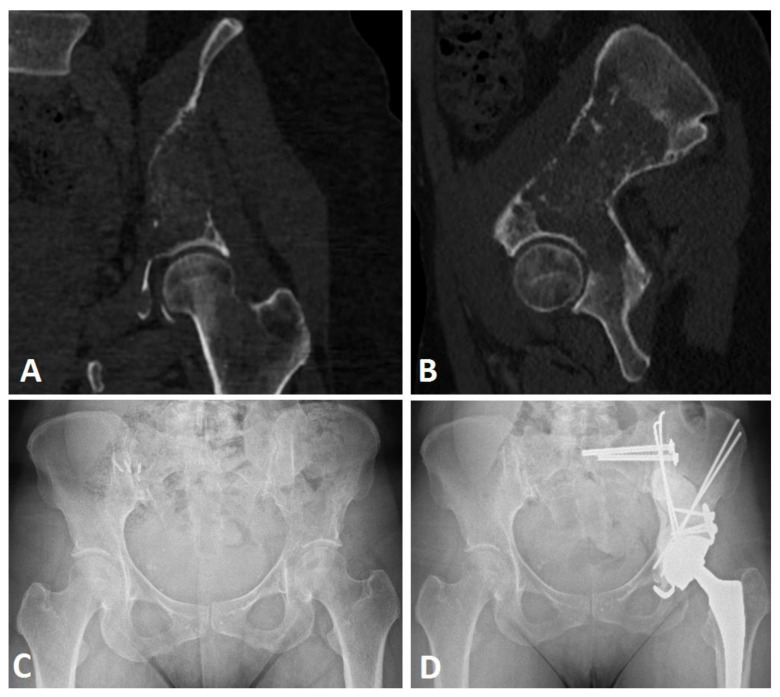
Harrington procedure (Steinman pins and cement) with THA with acetabular reinforcement using a Kerboul cross-plate and screws, and sacro-iliac joint screwing in a Harrington III lesion secondary to breast cancer. CT scan frontal (**A**) and sagittal (**B**), preoperative views, preoperative (**C**), and postoperative (**D**) AP X-ray views at 22 months of follow-up.

Patients were operated in a lateral position following a generic procedure by four senior surgeons specialized in tumor surgery in a single institution (Figure 1).

A conventional postero-lateral approach was systematically used to gain access to the iliac peri-acetabular metastatic lesion after femoral head section and extraction. After appropriate exposition, the curettage of the lesion was performed under direct vision to assess bone destruction and architecture, one exposure trochanterotomy was necessary (4.8%). After a secondary iliac crest incision, nonthreaded Steinman pins (2.5 mm, 3 mm, or 4 mm diameters) were used in an anterograde fashion from the anterior part of the iliac crest towards the posterior or medial wall, or from a more posterior origin aiming toward the anterior wall. Pins could also be inserted vertically, from the iliac crest tuberosity through the anterior gluteal line to the cavity [23]. The aim of the pin structure was to obtain sufficient stable conditions for acetabulum cementation. High viscosity cementation (Palacos, Heraeus, Germany) of the pins and the cavity was performed to complete the Harrington procedure, and lastly an acetabular component (Novae Stick/K; SERF, Décines, France) was sealed into the cement (Kerboul cross plate (Groupe Lepine^®^, Genay, France) (*n* = 18) and the stemmed acetabular cup (Integra, Groupe Lepine^®^, Genay, France) (*n* = 3)), aiming to restore the hip rotation center.

Finally, the femoral component was implanted in the standard manner. A dual mobility cup (Novae Stick/K; SERF^®^, Décines, France) was used systematically, aiming for 20° of anteversion and 40° of abduction. Postoperatively, patients were authorized for full weight-bearing with crutches or a walker depending on their general condition. Patients were systematically assessed clinically and radiologically at 3 months, 6 months, and 1-year follow-up.

### 2.3. Study Objectives

The main objective of the study was to (i) assess the joint function of patients undergoing a Harrington procedure with THA before and after surgery (at six and twelve months), using the Parker score and MSTS. Secondary objectives were to assess (ii) reoperation-free survival using the Henderson classification (iii) and overall survival rate, with an associated subanalysis for survival risk predictive score.

### 2.4. Statistics and Ethics

The patients’ data were collected retrospectively and included the counts and percentages for the qualitative variables and the minimums, maximums, means, standard deviations, and medians for the quantitative variables. The evolution of Parker and MSTS scores was analyzed using a Wilcoxon signed rank test. Reoperation-free survival and overall survival were described with a Kaplan–Meier curve. Deceased patients were censored by date of death. The alpha risk of all these tests was set at 5%, with a level of significance of *p* < 0.05. The data was collected using Microsoft^®^ Excel, the statistical analyses were performed using IBM^®^ SPSS Statistics V19 software (Armonk, New York, NY, USA), and the graphics were obtained using SAS software, version 9.4 (SAS Institute Inc., Cary, NC, USA).

The institutional review board approved the protocol. According to French legislation regarding anonymized data analyzed retrospectively (articles L.1121-1 paragraph 1 and R1121-2, Public Health Code), Nantes University Hospital has confirmed that approval from the ethics committee was not needed due to the noninterventional nature of the study, and thus no ethics committee approval was necessary at the time of the beginning of the study. The requisite processes were undertaken with the “Direction de la Recherche Clinique” (DRC) of the University Hospital of Nantes, France, and the “Commission Nationale de l’Informatique et des Libertés” (CNIL). The database was anonymized.

## 3. Results

### 3.1. Population Description

Our cohort consisted of 21 patients (Table 1), with a mean follow-up of 17.5 ± 14.9 months [min.: 0.1; max.: 64.6]. The most represented primitive tumors were breast (*n* = 7, 33.3%), pulmonary (*n* = 7, 33.3%), and renal cancer (*n* = 4, 19.0%). Acetabular lesions were mostly Harrington grade 3 lesions (*n* = 14, 66.7%). No major perioperative complications occurred in the cohort, nor any perioperative death.

**Figure 2:**
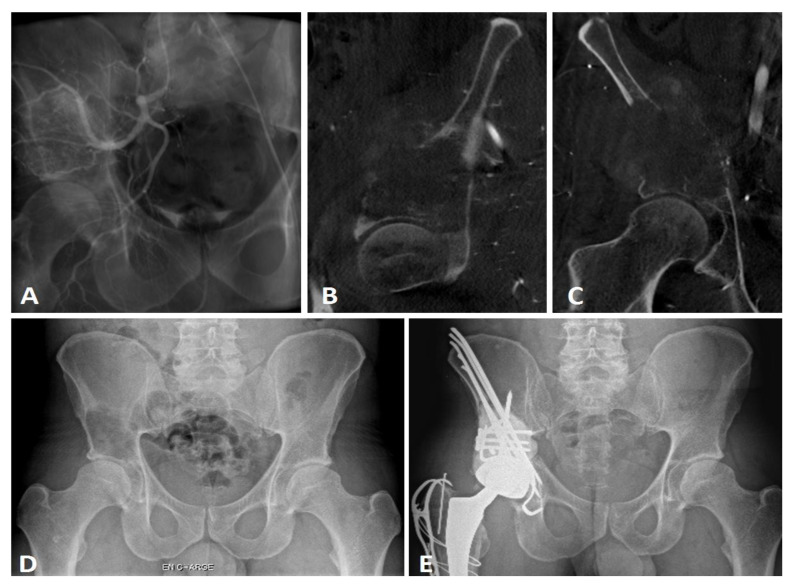
Imaging views of a GIST metastatic iliac lesion Harrington class III. Scout view (**A**) during the preoperative embolization, sagittal view (**B**) of the periacetabular lesion, frontal view (**C**). X-ray AP view of the pelvis before surgery (**D**), and postoperative X-rays at 18-month follow-up (**E**).

Before surgery, three patients had an embolization due to a renal clear cell carcinoma (*n* = 2, 14.3%) and GIST bone metastases (*n* = 1, 4.8%) (Figure 2). Perioperative X-ray control was performed in one case (4.8%), and associated sacro-iliac joint screwing were performed under O-ARM navigation for two cases (9.5%). Three patients were operated at a fractured stage (14.3%). All acetabular implants were a dual mobility cup, either in a Kerboul cross-plate (*n* = 18, 85.7%) or a stemmed acetabular cup (*n* = 3, 14.3%) with screws. The mean number of Steinman pins used was 3.0 ± 1.9 [min.: 1; max.: 8].

### 3.2. Parker and MSTS Evaluation

Parker score and MSTS were evaluated preoperatively in 21 patients, 12 at 6-month and 8 at 12-month follow-up (Table 2).

**Figure 3:**
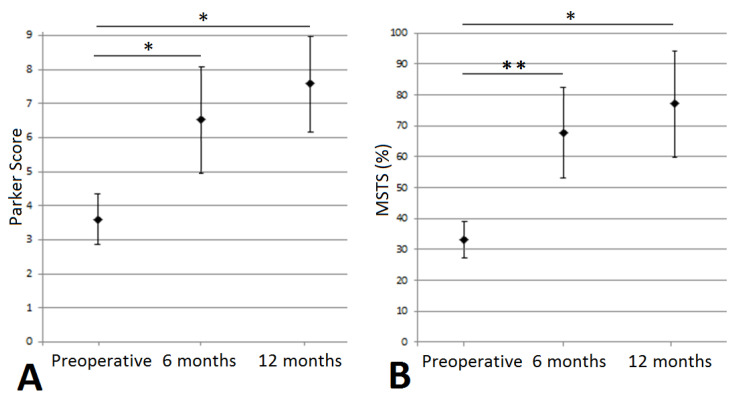
Functional assessment, preoperatively, at 6 and 12 months, (**A**) for Parker score and (**B**) for MSTS. *: [0.05; 0.01], **: [0.01; 0.001].

The mean Parker score values were significantly higher at 6 months (6.6 ± 3.2, *p* = 0.049) and 12 months (7.6 ± 2.1, *p* = 0.028) postoperatively compared to preoperatively (3.6 ± 2.0) (Figure 3A). There was no significant difference between postoperative scores at 6 and 12 months (*p* = 0.176).

For the MSTS score, we observed significantly higher mean scores at 6 months (67.0 ± 30.6, *p* = 0.008) and 12 months (82.4 ± 24.0, *p* = 0.017) postoperatively compared to preoperatively (32.2 ± 16.2) (Figure 3B) (Table 1). There was no significant difference between postoperative scores at 6 and 12 months (*p* = 0.175).

### 3.3. Reoperation-Free Survival

**Figure 4:**
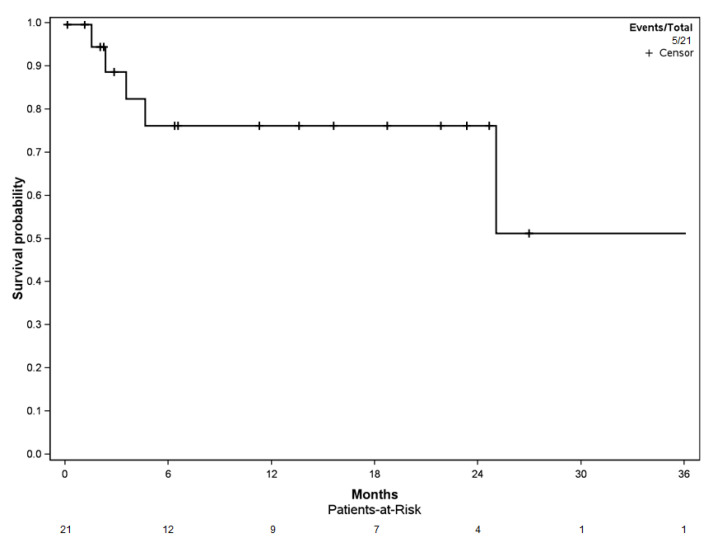
Kaplan–Meier curve for reoperation-free survival.

Reoperation-free survival among the cohort was 76.1% [CI 95%: 58.1–99.7] at the 6-, 12-, and 24-month follow-up (Figure 4). Four of the five reoperation events (80.0%) occurred in the first 6 months of follow-up.

There were no intraoperative deaths or major complications such as vascular or nerve injuries and excessive bleeding. We observed one death at postoperative day 3 due to a massive pulmonary embolism. A total of 12 complications occurred in five patients (all of them Grade 3 Harrington lesions), the most frequent of them being pin migration (50%, *n* = 6), then infections (20.0%, *n* = 3), seroma (16.7%, *n* = 2), and acetabular loosening (8.3%, *n* = 1) secondary to multiple pin migration. All these complications led to reoperations (Table 3). Two of the five patients had more than one complication (40.0%): one had four consecutive complications consisting in a recurrent massive postradiation therapy seroma leading to infection and pin migration. The second presented five complications with two consecutive pin migrations, leading to acetabular loosening and recurrent infection. Of the twelve reoperations, six (50.0%) involved a pin removal or pin recut, three debridement antibiotics and implant retention (DAIR) (25.0%), one an acetabular revision (8.3%), one seroma drainage (8.3%), and one single stage complete revision in a septic context (8.3%).

### 3.4. Patient Overall Survival

**Figure 5:**
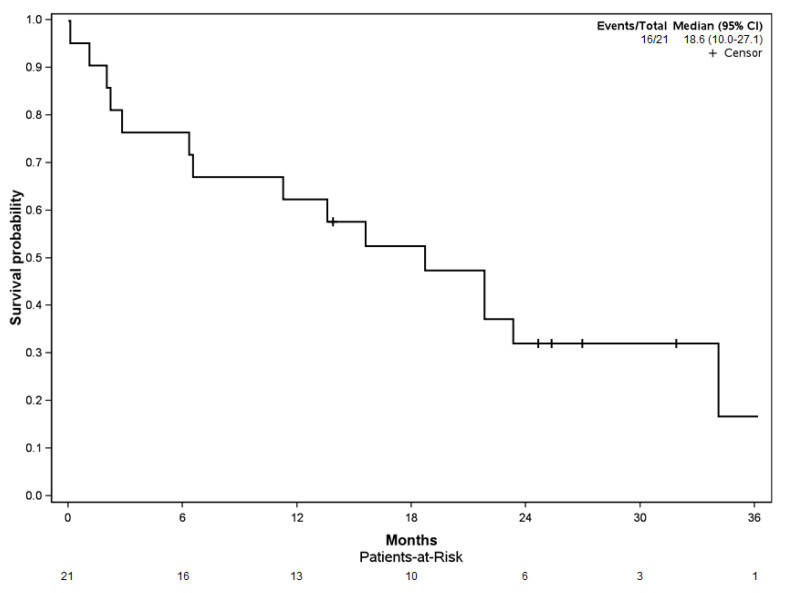
Kaplan–Meier curve for patient overall survival.

The probability of patient survival was 76.2% [95% CI: 59.9–96.7] at 6 months, 61.9% [95% CI: 44.3–86.6] at 12 months, and 31.2% [95% CI: 16.2–60.0] at 24 months (Figure 5). The median overall survival rate of patients within the cohort was 18.6 months [95% CI: 10.0–27.1].

The Katagiri score was evaluated for each patient in the cohort, accounting for six patients with low-risk (28.6%), thirteen with intermediate-risk (61.9%), and two with high-risk (9.5%) (Table 4). The median survival after the Harrington procedure was 33.9 months [95% CI: 17.9–50.0] in the low-risk group, 11.2 months [95% CI: 0.0–25.1] in the intermediate-risk group, and 6.5 months in the high-risk group (no confidence interval due to the small number of patients).

**Figure 6:**
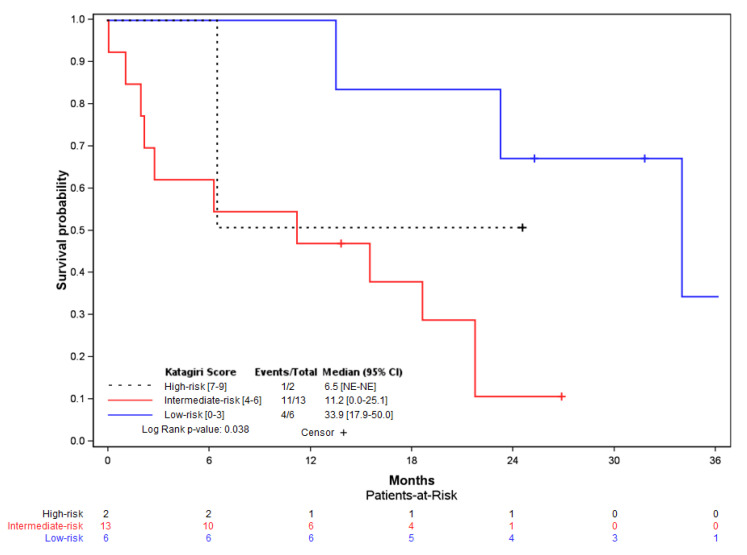
Kaplan–Meier curve for patient overall survival depending on the Katagiri risk score, with log-rank analysis (*p* = 0.038).

Subanalysis on the Katagiri scores and patient overall survival showed a significant difference between the high- (*n* = 2), intermediate- (*n* = 13), and low-risk patients (*n* = 6) (log-rank, *p* = 0.038) (Figure 6).

## 4. Discussion

**Figure 7:**
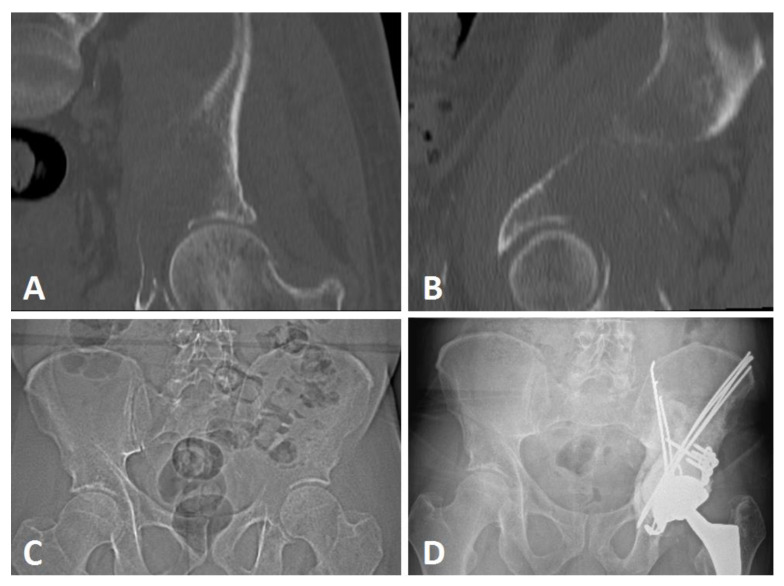
Imaging views of a renal clear cell carcinoma metastatic iliac lesion Harrington class III. Frontal view (**A**) of the periacetabular lesion, sagittal view (**B**), X-ray AP view of the pelvis before surgery (**C**), and postoperative X-ray (**D**) at 22-month follow-up.

**Figure 8:**
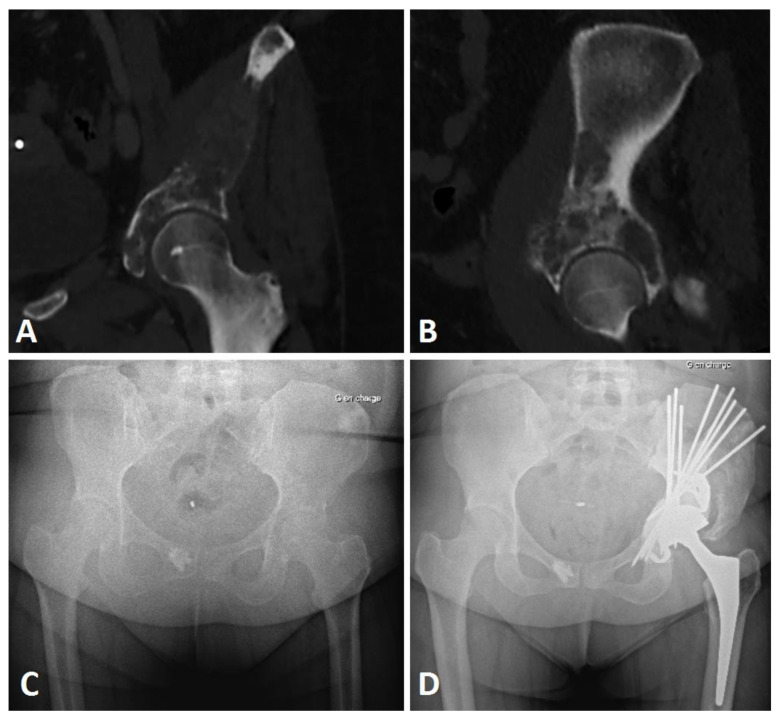
Imaging views of a breast metastatic iliac lesion Harrington class III. CT scan frontal (**A**) and sagittal views (**B**), preoperative AP X-ray (**C**), and postoperative (**D**) views of a Harrington procedure (Steinman pins and cement) associated with THA with acetabular reinforcement using a Kerboul cross-plate, in a metastatic localization of breast cancer with pelvic discontinuity at 2-year follow-up, Parker 9.

Extensive metastatic lesions of the acetabulum may be associated with severe disability and pain. Patients should be considered for surgery if nonoperative treatment options such as local radiotherapy, bisphosphonate, and/or hormonal therapy or partial weight-bearing do not result in sustained improvement of the situation [17]. The Harrington procedure associated with THA is a simple, inexpensive, and versatile technique, which is still in use nowadays for extensive bone loss lesions in complex cases [13,24]. Indications still remain despite the use of other implants such as stemmed acetabular cups or reinforcement rings [14,25,26]. The anterograde pinning technique we described makes it possible to bridge acetabular defects from zone I to zone III (Enneking), mostly in Harrington class 3 (66.7%) or complex class 2 cases (32.3%). The procedure could thus restore the roof and medial wall, but also anterior and posterior wall supports; it is a last-option indication in pelvic discontinuity or associated iliac isthmus destruction (Figure 7 and Figure 8). In our practice, we do not use this procedure for Harrington class 4 lesions; in this situation, we mostly perform acetabular reconstruction using a massive stemmed acetabular cup.

It should be noted that, in our practice, we reserve the Harrington procedure with anterograde pinning from the iliac crest for patients in whom there is not enough bone support to use a single device (standard Kerboul cross-plate or a stemmed acetabular cup) [26], as a conventional cemented acetabular cup is rarely a valid option, especially after radiation therapy, associated with higher loosening rates [27]. The secondary approach from the iliac crest [23] makes it possible to aim the pins for medial, anterior, and posterior wall restitution. Other authors propose retrograde pinning from the ischial tuberosity [28], and Chimutengwende-Gordon et al. also add to this construction an intrapelvic suprapectineal plate for anterior column restitution.

This bridging structure is difficult to perform using a single approach in our experience with only screws from the acetabulum defect or implant.

The second approach on the iliac crest described in our procedure might expose patients to specific complications, longer surgery, and potential bleeding that might impair restoration of function; and even if the Harrington procedure is simple and versatile, there remains a lack of functional results assessing this procedure. Our study thus provides interesting data with significant functional improvements both on Parker and MSTS scores.

### 4.1. Functional Assessment

Within the cohort, the functional results showed a significant improvement, as early as 6 months after the operation, with the 12-month functional assessment showing the best results (Figure 7 and Figure 8). The MSTS score improved from 32.2% to 67.7% at 6 months, and to 82.4% at 12 months. The improvement was also significant on the Parker score; from 3.6 preoperatively to 6.6 at 6 months, and 7.6 at 12 months following the Harrington procedure associated with THA. We observed a continuous improvement in patients still alive at 12 months on both these scores. These functional results are in line with the study by Ho et al., where improvements in the MSTS score were seen at 4 months following a modified Harrington procedure in a single approach fashion [15]. For modified Harrington procedures, Kiativesi et al. described in 22 patients a mean MSTS score of 70% [29], and Erol et al. in 16 patients obtained a mean MSTS score of 72% postoperatively [30], which is similar to ours.

In our study, we corroborate the advantages of the Harrington surgery for functional recovery in patients with iliac metastatic localizations. The functional analysis was performed retrospectively: MSTS and Parker scores were generated by a single investigator, thus limiting any potential bias; a recent report has underscored the reproducibility of MSTS scores based on medical records, with a slight inherent risk of overestimation [31]. Subsequent prospective works focusing on this topic need to associate more clinical specific evaluations, with dedicated hip scores, or as the 6 min walk test (6MWT) [32].

### 4.2. Reoperation-Free Survival and Complications

The reoperation-free survival rate was 76.1% at 6 and 12 months. These results were in line with the literature on this subject. Most of the first revision events occurred during the first 6 months of follow-up (80%). From the five patients with complications, two account for nine (75.0%) of the twelve complications, with consecutive surgeries in frail patients. On the other hand, the other three patients were operated on for a single pin migration.

Pin mobilization was the most frequent complication in our cohort, accounting for half of the whole count. It is a specific complication reported by various authors [33,34]. The reoperations following these were minor, consisting in a simple pin removal. Nonetheless, it might expose patients to septic issues, due to potential skin perforations, as seen in one of our patients, or even acetabular aseptic loosening. These reoperations must be avoided as much as possible in metastatic patients, to preserve the patients’ quality of life and adjuvant treatment administration. Therefore, regarding our results, and as suggested by de l’Escalopier et al. and others [13,23,34], we recommend using threaded pins instead of unthreaded Steinman pins to reduce this specific complication risk; and we since have modified our practice on this point.

Other complications identified in the cohort were infection (25.0%), seroma (16.7%), or acetabular loosening (8.3%). These complications are classically reported in the literature for this type of surgery [15,35]. In a recent work by Chang et al., a complication rate of 30% was observed, similar to that of our cohort [24]; in the same way, Erol et al. identified 75% implant survival, reporting a 6.3% dislocation rate [30]. Kask et al., in a cohort of 89 patients, identified eight dislocations using conventional and constrained cups [36], and Bagsby et al. reported a 21.0% dislocation rate compiling 185 patients from various studies using the Harrington procedure [35]. We did not find any dislocation in our cohort, whereas it is reported to be the main complication in most series [14,37]. We think this might be linked to the systematic use of dual mobility sockets in our series, reducing the risk of dislocation, as highlighted in previous works [13,26,38,39,40].

### 4.3. Overall Survival and Katagiri Score

We observed a 6-month overall survival of 76.2%, and a 12-month survival of 61.9% in our cohort, where the median overall survival was 18.6 months. This median overall survival is higher than the 8 months described by Kask et al. and may be due to patient selection differences [36]. For the Harrington procedure indication, multidisciplinary teams must take into the account the patients’ overall health status and oncologic prognosis besides the anatomic location and extent of the disease [41]. A precise prognosis analysis is mandatory. The Katagiri predictive score was used in our daily practice, and other efficient tools such as PATHFx or OPTModel have since been developed for this purpose [42,43,44].

We used the latest version of the Katagiri score in our study to maintain comparability [21], except for the primary tumor growth characteristics, for which we used the 2005 version on this item for patients operated before 2014 [20]. Due to progress in treatments, it seemed more precise to differentiate this item, as some forms of cancer have seen their prognosis change radically in the last 20 years. The Katagiri risk-group was significantly associated with survival probability in our cohort (*p* = 0.038), as shown by various studies [20,21,45,46], with higher median overall survival rates in the low-risk group (33.9 months) compared to intermediate-risk (11.2 months) and high-risk (6.5 months). As the Harrington surgery may mostly benefit patients with survival of more than 6 months, with a higher expected survival, it is necessary to use reliable prognostic tools. In this aspect, the Katagiri score, which has not been evaluated before in this indication, seemed to be efficient in our study despite the small cohort, following a complex surgery such as the Harrington procedure associated with THA.

### 4.4. Limitations and Strengths

We can admit potential bias due to the retrospective design of our study and our small cohort on a rare subject. The patient identification procedure may represent a selection bias, as we cannot guarantee absolute completeness. In addition, our analysis may show some flaws with data missing for a few items (preoperative laboratory results, perioperative blood loss, and postoperative radiation therapy). Another limitation of this study is its retrospective nature without a control group, which is difficult to use due to the rarity of the disease and the lack of surgical alternatives in Harrington 3 bone defect surgical procedures.

Despite our small cohort, we highlighted a high functional benefit of the Harrington procedure in complex extensive bone loss. We also emphasize the choice of threaded Steinman pins in the Harrington procedure, due to the risk of migration. Moreover, our work revealed a low dislocation rate using dual mobility sockets in these surgeries with a high risk of postoperative instability, and we confirm work from others on this point [13,40].

## 5. Conclusions

This study highlights the significant functional improvements following the Harrington procedure, despite a non-negligible reoperation rate, dominated by pin mobilization. Dedicated threaded pin devices can be used to decrease this specific complication. Moreover, the use of dual-mobility acetabular cups in these cases is associated with low dislocation rates. The Harrington procedure fulfilled its objective for functional improvement in a metastatic context, and the Katagiri prognosis score seemed efficient on this indication for helping with on decision-making in these complex cases.

## Figures and Tables

**Table 1:** Population characteristics.

			Total, *n* = 21
Demographic data		Sex ratio (M/F)	10/11
		BMI (kg/m^2^)	24.4 ± 5.4
		Age (years)	60.7 ± 13.0
	ASA Score	ASA 2	10 (47.6%)
		ASA 3	11 (52.4%)
	Comorbidities	Active smoker	3 (14.3%)
		Obesity	2 (9.5%)
		COPD or asthma	2 (9.5%)
		Cardiac condition	3 (14.3%)
		Diabetes	3 (14.3%)
Oncologic data		Pathologic fracture	3 (14.3%)
	Primitive tumor	Breast cancer	7 (33.3%)
		Pulmonary cancer	7 (33.3%)
		Renal carcinoma	4 (19.0%)
		Prostatic cancer	1 (4.8%)
		GIST cancer	1 (4.8%)
	Harrington Grade	Grade 2	8 (32.3%)
		Grade 3	14 (66.7%)
	Enneking Zones	Zone 1	10 (47.6%)
		Zone 2	21 (100%)
		Zone 3	3 (14.3%)
Associated treatments	Radiation therapy	Preoperative	8 (38.1%)
		Postoperative (*n* = 18)	14 (77.8%)
	Chemotherapy	Preoperative	16 (76.2%)
		Postoperative	17 (81.0%)
		Preoperative embolization	3 (14.3%)
Surgical Data		Surgery duration (minutes)	135 ± 29
		Blood loss(mL) (*n* = 13)	1433 ± 1177
		RBCs Transfusion (mL)	982 ± 726
		Steinman pins number	3.0 ± 1.9
	Associated gesture	Trochanterotomy	1 (4.8%)
		Sacro-iliac joint screwing	2 (9.5%)
	Acetabular implant	Kerboul cross-plate	18 (85.7%)
		Stemmed acetabular cup	3 (14.3%)

BMI: Body mass index; ASA: American Society of Anesthesiologists; COPD: Chronic obstructive pulmonary disease; GIST: Gastro-intestinal stromal tumor; RBC: Red blood cell.

**Table 2:** Functional score assessment with Parker score and Musculoskeletal Tumor Society score following Harrington procedure.

		Parker Score (%)	MSTS Score (%)
Preoperative (*n* = 21)	Mean ± SD	3.6 ± 2.0	32.2 ± 16.2
	Median [Min; Max]	4.0 [0.0; 9.0]	31.6 [6.7; 66.6]
6 months (*n* = 12)	Mean ± SD	6.6 ± 3.2	67.8 ± 30.6
	Median [Min; Max]	8.0 [0.0; 9.0]	78.4 [3.3; 100.0]
12 months (*n* = 8)	Mean ± SD	7.6 ± 2.1	82.4 ± 24.0
	Median [Min; Max]	9.0 [4.0; 9.0]	89.9 [30.0; 100.0]

MSTS: Musculoskeletal Tumor Society; SD: Standard deviation.

**Table 3:** Description of the complications.

		Total, *n* = 12
Delay between surgery and complication (months)	Mean ± SD	12.4 ± 11.9
	Median [Q1; Q3]	5.2 [1.6; 30.7]
Complication type	Pin migration	6 (53.9%)
	Infection	3 (25.0%)
	Seroma	2 (16.7%)
	Acetabular loosening	1 (8.3%)
Henderson Classification	1	2 (16.7%)
	3	6 (50.0%)
	4	3 (25.0%)
	5	1 (8.3%)

SD: Standard deviation.

**Table 4:** Katagiri score evaluation of survival [20,21].

		Score	Total, *n* = 21
Primary tumor growth characteristics	Slow	0	9 (42.9%)
	Moderate	2	6 (28.6%)
	Rapid	3	6 (28.6%)
Visceral metastases	No	0	14 (66.7%)
	Nodular	1	0 (0.0%)
	Disseminated	2	7 (33.3%)
Laboratory data * (*n* = 13)	Normal	0	7 (33.3%)
	Abnormal	1	4 (19.0%)
	Critical	2	2 (9.5%)
ECOG PS	0–2	0	13 (61.9%)
	3–4	1	8 (38.1%)
Previous chemotherapy	Yes	1	16 (76.2%)
	No	0	5 (23.8%)
Multiple skeletal metastases	Yes	1	19 (90.5%)
	No	0	2 (8.5%)
Katagiri Score	Low-risk	(0–3)	6 (28.6%)
	Intermediate-risk	(4–6)	13 (61.9%)
	High-risk	(7–10)	2 (9.5%)

ECOG PS: Eastern Cooperative Oncology Group Performans Status. * Missing laboratory data were given a 0 value.

## Data Availability

The datasets generated and/or analyzed during the current study are available from the corresponding author on reasonable request.
